# What Makes a Nobel Prize Innovator? Early Growth Experiences and Personality Traits

**DOI:** 10.3389/fpsyg.2022.845164

**Published:** 2022-03-10

**Authors:** Linlin Zheng, Yenchun Jim Wu, Yuyi Li, Di Ye, Wenzhuo Li

**Affiliations:** ^1^Business School, Huaqiao University, Quanzhou, China; ^2^College of Humanities and Arts, National Taipei University of Education, Taipei City, Taiwan; ^3^Graduate Institute of Global Business and Strategy, National Taiwan Normal University, Taipei City, Taiwan; ^4^Business School, HoHai University, Nanjing, China

**Keywords:** early growth experience, personality traits, configuration matching, original innovation, science and technology, Nobel Prize

## Abstract

The original innovation talents and their achievements promote the development of natural science and are regarded as a symbol of the national comprehensive power. This study explores the process that causes original innovation talents’ personality, uses fuzzy-set qualitative comparative analysis, and explores the linkage between configurations made up of early growth experiences and personality. We took Nobel Prize winners as samples and discovered that high responsibility was inspired by high family democracy driving, high family size driving, high family function driving, and high teaching democracy driving; high extroversion was inspired by high family size driving, high family democracy driving, and high family status driving; high openness was inspired by high family status driving, high family democracy driving, high family size driving, both high open teaching and educational level driving, as well as high peer support driving; high or non-high family status brought high extroversion or openness; non-high teacher accomplishments and teacher-student relationships produced high openness; non-high extroversion came with non-high teacher-student relationship. We proposed strategies for strengthening the positive effects or avoiding the negative effects of early growing-up experiences on personality.

## Introduction

Original innovation talents are individuals who have made great discoveries or inventions in natural science and technology (Bartneck, 2008). Moreover, talent scale and hierarchy determine a nation’s scientific and technological trends (National Research Council et al., 2010; Silvanto and Ryan, 2018). Since the implementation of the Nobel Prize Plan in 2001, Japan has produced on average one Nobel Prize winner a year (Kapranov, 2019). Tu Youyou, a Chinese native scientist who was on the list of world famous scientists in 2015, made her artemisinin discovery in 1972 and achieved the first Nobel Prize in natural science for China (Thomas et al., 2016). At least 20 academicians who have made outstanding contributions to China’s advances in science and technology passed away in 2019. Given this, it can be seen that the inadequate reserves and aging phenomenon of top talents in technology and science make it imperative to consolidate the foundation of original innovation talents. Using the growth rules of the talent pool to carry out a talent cultivation plan has become a key idea. In the past, research on original innovation talents focused on the common growth experience. At the earliest, the American sociologist Harriet Zuckerman analyzed Nobel Prize winners’ family backgrounds and other experiences (Zuckerman, 1977). There are few empirical studies that take the early growth experience as the antecedent of personality, and the conclusion drawn through traditional marginal analysis loses touch with reality. Scholars have discussed the growth of original innovation talents from the single environmental perspective and ignore the integrity of various experiences and the complementarity of other experiences (Rothenberg, 2005; Hensley, 2009). Research has not established a comprehensive and logical framework for an early growth experience. In this study, we considered that the individual’s personality traits are complex under the synergistic influence of different growth experiences. Since only one of the experiences can intervene and change the overall essence of the growth process, an individual’s growth has high uncertainty (Clarke, 2002; Csikszentmihalyi, 2010).

Based on this, the early growth experience was summed up in various configurations, and fsQCA was used to investigate which configuration of early growth experience produced high or non-high personality traits. We attempt to answer the following questions: What are the driving paths for their personality traits? Which path constrains personality traits? The answers to the above questions provide the basis for training original innovation talents.

## Literature Review

### Early Growth Experience and Personality Traits

#### Family Experience

Emotional warmth and behavioral guidance create a safe, liberal, and democratic environment (Pereira et al., 2008), which helps individuals to accept the outside world, to make independent decisions (Tennent and Berthelsen, 1997; Sen and Sharma, 2013) and to be more composed, responsible, prosocial, and grateful (Kempel, 2009; Hensley, 2009). Positive parenting predicts openness and promotes conflict resolution. In comparison, punishment or overprotection leads to indifference, aggression, and adventure. Parental support has a greater impact on individual self-awareness and self-control than parenting style (Bryant et al., 2006; Lancee, 2018). It is difficult for parents with low socioeconomic status to implement democratic parenting (Corwyn and Bradley, 2002). Based on the theory of family status realization, the higher the parents’ occupational status and educational level, the stronger the individual’s desire to pursue further education, to try non-traditional careers, and to stimulate achievement sense and openness (Rothenberg, 2005). Personality is also related to family size (Lee et al., 2006); therefore, siblings may crowd out family resources, and the order of birth and emotional distance of siblings influence individual growth (Clark and Rice, 2010).

#### School Experience

Both Chinese academicians and Western scientists have higher educational experiences (Youtie et al., 2013; Chauhan et al., 2018), and universities play an important role. Accordingly, young Yangtze River scholars are influenced by the sequential, advanced levels of the master’s and doctoral degrees, studying at more than one school, pursuing overseas educational and post-doctoral experiences (Kempel, 2009; Su, 2009). The overseas education allows them to apply for an advanced achievement with high risk, which will accelerate their career promotion (Wang, 2013). The growth of scientists is also related to excellent tutors and tutor achievements (Kaewviset et al., 2015), which cannot be separated from the dialog and mutual understanding between teachers and students; their life ideals are influenced by the school and the relationship between teachers and themselves in their youth (Southerland and Bahbah, 2011).

#### Working Experience

The success of young scientific and technological innovators is related to their work experience, which involves leadership support, work content, interpersonal relationships, funds and facilities, and training (Abbott, 2006). The innovation atmosphere of an organization is divided into team cooperation, supervisory, and organizational support (He et al., 2020, 2021). Talent growth is faster when individual values and goals are closer to the organization’s values and goals (Hull, 1978) and when organizational commitment, job performance, and job satisfaction of individuals are higher (Law et al., 2007). Visible organizational support indirectly affects employees’ innovative behavior (Saether, 2019). Moreover, organizational social responsibility positively predicts personal relationships and work experience, which is important for scientific and technological talents (Brockelman, 2007).

This study refined the factors’ dimensions, which influence the personality of original innovation talents from family, school, and the workplace. It constructed a highly generalized framework for original innovation talents’ personalities based on the Big Five personality traits theory and endowed them with original innovation characteristics. We regarded the early growth experience as a whole and discussed its influence on personality traits. It is unreasonable to judge that certain personality traits can only be enhanced or weakened by a single dimension of growth experienced by traditional marginal analysis, so it is necessary to conduct a complex causal analysis on the configuration effect on personality traits.

## Research Design

### Index System

In addition to the previous literature review, some scholars’ theories also lay the foundation for the classification of early growth experience. Personality stereotype of original innovation talents is highly related to the cultivation of personality traits in adolescence. Therefore, the early growth experience in our study is mainly positioned in the experiences which occur in the golden period when an individual is a child or youth. The living environment of original innovation talents is inseparable from family and school (Sternberg and Lubart, 1993). For example, the family is an important microsystem for individual development (Bronfenbrenner, 1986). According to the personality development theory proposed by Erikson, parents are involved in the first five stages of personality development, which goes through eight stages, and various stages are attached to one another. Facts prove that original innovation talents can withstand loneliness and success, owing to the support of spouses and other members in the family, or the practical assistance of friends and partners in the work (Harrington, 2011). The classification indexes of the early growth experience of the original innovation talents are listed in Table 1.

**TABLE 1:** Indexes about early growth experiences of original innovation talents.

First-level index	Secondary index
Family experience	Family democracy
	Family size
	Family status
	Family function
	Family support
School experience	Teacher accomplishments
	Democracy teaching
	Open teaching
	Teacher-student relationship
	Peer support
	Education level
Working experience	Organizational support
	Colleague relations

This study used the Big Five personality as a measuring tool due to the following advantage: it covers almost all personality categories and is honored as “the ocean of personality” by believers. It can be used to comprehensively and systematically reflect the various characteristics of original innovation talents. It has also been revised and improved for a long time and gradually given a more clear primary semantics. Many psychologists believe that the Big Five personality is the best model bred in Western culture, with cross-culture and cross-region advantages. The Big Five personality and the original innovation theory imply and emphasize the innovation potential.

For example, a series of innovative traits in extroversion are mentioned. Passion is the driving force of creativity (Beard, 2012). Original innovation talents have team awareness, independent judgment. They accept risks, do not follow the public, and are often energetic, calm, and confident (Torrance, 1962; Barron, 1969; Dacey and Lennon, 1998). A series of innovative traits in responsibility are mentioned in the research of original innovation talents, including strong will, strong ambition, lack of fear of failure, cautiousness, and arrangement. A series of innovative traits in openness are mentioned in the research of original innovation talents, including imagination, aesthetic, observation, strategic vision, association, and critical thinking (Torrance, 1962; Guilford, 1968; Sternberg and Lubart, 1993), as well as open experience, new try, and new rules (Simonton, 1984; Verganti, 2008). A series of innovative traits in the agreeableness are mentioned in the research of original innovation talents, including altruism, dedication, and consensus with team members (Reid et al., 2014). A series of innovative traits in extroversion have been mentioned in the research of original innovation talents, including crazy inspiration, anxiety, and depression (Torrance, 1962; Averill et al., 2001).

Therefore, the Big Five personality can provide a broad framework for the description and division of the personality traits of original innovation talents (Table 2).

**TABLE 2:** Semantic framework for the conceptual model of personality traits of original innovation talent.

Personality traits	Semantic keywords
Imagination	Surreal transformation of knowledge or objects stored in memory
Aesthetic	Appreciate the beauty of things or art, and integrate the pleasant feeling into innovation activities
Rich feeling	Good at association and sharp intuition, and make inferences about the development tendency or basic rules of things
Try something new	Tolerate the collision of new ideas from others and prefer novelty and diversity
Speculation	Be good at thinking and debating, constantly find and solve problems, and dialectically reveal the internal relationship between things
Values	Pursue an objective and fair attitude in scientific research, question or challenge authority or conventional opinion
Competency	Grasp the overall direction, have the ability to carry out the work independently
Well organized	Arrange research in a planned and orderly way
Conscience	Seeking the balance between science and ethics
Achievement	High expectation of research results and success, strong ambition and sense of goal
Self-discipline	Not afraid of long-term original innovation process, overcome the difficulties and focus on the research process
Cautiousness	Pay close attention to external things or people’s words and deeds, and be responsible for the authenticity and effectiveness of research results
Passion	Strict and friendly working condition, high working enthusiasm
Cooperation	Cooperate with others or seek extensive contacts in scientific research
Arbitrariness	Put forward independent opinions, stick to correct ideas in conflict with others or authority
Vitality	Fast pace of life, seeking other means to relax after experiencing high-intensity scientific research activities
Seek stimulation	Dare to take risks in high-risk original practice
Positive emotions	Keep happy, optimistic and confident when scientific research is in trouble
Anxiety	When there is no progress in scientific research or the problem cannot be solved, can’t eat and sleep well
Angry and hostile	Hate pseudoscience, resist wrong and outdated scientific research conclusions, and admit mistakes and injustice
Depression	Don’t have the heart to do other things and feel unhappy when encounter difficulties or can’t find a solution
Self-awareness	Emphasize personal opinions and don’t listen to others’ persuasion
Impulse	Keep inspiration in mind and pursue quick action to avoid missing opportunities and put ideas into practice immediately
Vulnerability	The mood fluctuates in the difficult situation, affecting the scientific research work
Trust	Believe in the ability and quality of others, entrust their own important affairs to others, and personally impart scientific research experience
Sincerity	Direct, frank, honest, without reservation of knowledge
Altruism	Protect others and make a self-sacrifice in an extremely dangerous scientific research environment
Compliance	Obey the organization and accept criticism or other people’s opinions with an open mind
Humility	Do not be arrogant or hypocritical because of academic status and academic resources
Empathy	Have resonance and understand other team members in scientific research

### Data Sources and Processing

A total of 194 Nobel Prize winners were selected as research samples. The selected criteria are as follows: the winners’ personal experience has been tracked for many years, so it is easy to avoid distorted or incomplete information. The winners’ research field focused on physics. Since physical discoveries and inventions promote the experimental discoveries of other disciplines such as chemistry and biology, social productivity, people’s livelihood, and national defense. The scores of early growth experience and personality traits were obtained using the biographical method which objectively reflects the development track of original innovation talents to grow into outstanding talents, and data were collected using the official Nobel Prize website. A Likert scale was used to rate the growth experience and personality. The reliability analysis and validity analysis are shown in Table 3.

**TABLE 3:** The reliability analysis and KMO and Bartlett’s test.

		Personality	Early growth experience
Kaiser-Meyer-Olkin		0.879	0.822
Bartlett’s Test	χ2	3628.080	463.713
	df	435	78
	Sig.	0.000	0.000
Cronbach’s Alpha		0.92	0.765

This study conducted a confirmatory factor analysis on the conceptual models of personality and early growth experience, respectively. The results shown in Table 4 reveal that the main indexes were excellent, and the relationship between variables could be further tested.

**TABLE 4:** Model fit summary.

	χ*^2^*	*df*	χ*^2^/df*	*CFI*	*IFI*	*RMSEA*
Big five personality	777.633	345	2.254	0.933	0.933	0.058
Early growth experiences	35.071	62	0.566	0.99	1	0.000

**FIGURE 1:**
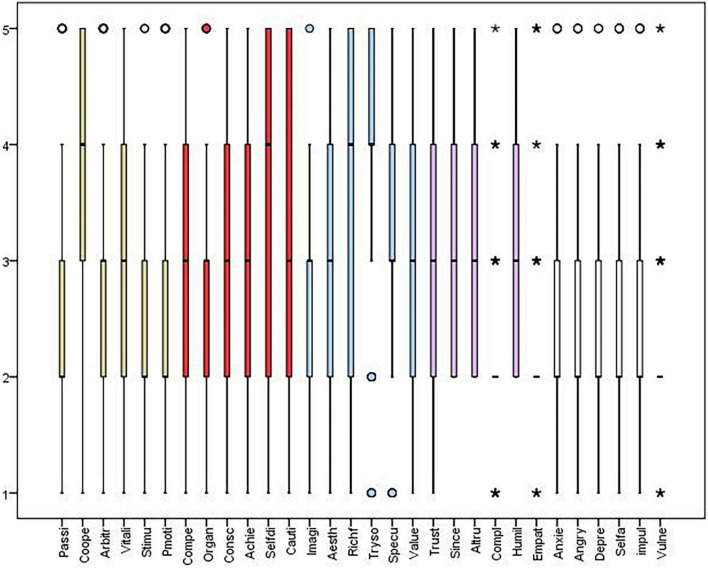
Boxplot of personality score. “*” indicates outlier of personality score.

As shown in Figure 1, the Nobel Prize winners showed more tendencies of openness, responsibility, and extroversion in original innovation, and the most significant personality traits of them were trying something new, self-discipline, cooperation, and humility.

### Research Method

Since the emergence of personality traits of original innovation talents depends on the configuration that consists of various experiences which are interrelated and interact with each other, rather than a certain experience that directly determines a certain personality. fsQCA has made some interesting contributions to perfect management practice with consideration of interdependent conditions, handles condition configuration analysis, and analyzes various possibilities of a given result. At the same time, it holds that causality is asymmetric, which determines that the causes of high and non-high personality traits cannot be reversed. Overall, fsQCA also overcomes the limitations that exist in the traditional marginal analysis. For example, findings cannot be generalized, and quantitative analysis cannot be sufficient to explain all cases. The traditional marginal analysis only considers the relationship between a single independent variable and dependent variable and thinks that a certain condition directly determines the personality level.

## Results

### Antecedent and Outcome

**FIGURE 2:**
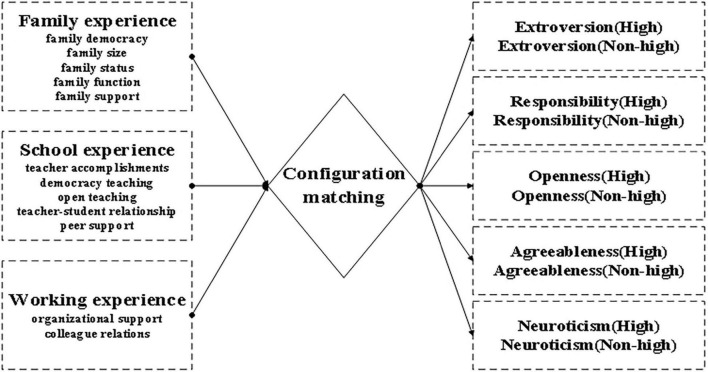
Configuration matching model.

The fsQCA research model is shown in Figure 2.

(1) Antecedent variable. The antecedents include secondary indicators of family experience, secondary indicators of school experience, and secondary indicators of work experience.
(2) Result variable. Five personalities, such as extroversion, responsibility, openness, agreeableness, and neuroticism, were taken as the outcome variables influenced by the early growth experiences.

### Calibration and Reassignment of Variable

Variable value was calibrated into a score from 0 to 1. After calibration, the scatter distribution of the fuzzy-set data and original data showed that the score assignment was reasonable.

### Necessity Test

Before the truth table analysis, we checked that the necessity of an antecedent influencing high or non-high personality traits by a single condition did not exceed 0.9. Therefore, these conditions were incorporated to further explore configuration effects on personality traits.

### Configuration Analysis Result

In configuration analysis, the identification of core conditions and secondary conditions in each configuration was obtained. The core condition refers to the condition that has an important influence on the result, and the secondary condition refers to the condition that plays a secondary role in the generation of a result. The black circles indicate the presence of a condition, circles with × indicate its absence, large and bold circles indicate core condition, the small or thin circle indicates secondary condition, and blank spaces indicate “don’t care.” Specifically, the small black circle indicates the presence of a secondary condition, the big black circle indicates the presence of a core condition, the thin circle with × indicates its absence of a secondary condition, and the bold circle with × indicates its absence of a core condition. The first two letters of the abbreviation in the title line of Tables 5–10 represent an acronym for two words about personality and experience, and the last number of the abbreviation represents the serial number of the corresponding configuration solution.

**TABLE 5:** Matching analysis of family experience and high original innovation personality.

Antecedents	Extroversion configuration solution		Responsibility configuration solution		Openness configuration solution			
	EF1	EF2	RF1	RF2	OF1	OF2	OF3	OF4
Family democracy	⊗	●	●		⊗	⚫	⚫	⊗
Family size	●	⊗	⊗	●		⊗	●	⚫
Family function	⊗	⊗		●	●		●	●
Family status	⊗	●	⚫	⚫	⚫	●		⊗
Family support	⚫	⚫	⚫	⚫	⊗	●	●	●
Consistency	0.8	0.8	0.8	0.8	0.9	0.9	0.8	0.9
Original cover degree	0.2	0.2	0.3	0.5	0.2	0.3	0.5	0.1
Unique cover degree	0.1	0.1	0.1	0.3	0.1	0.1	0.2	0.01
Total consistency	0.8		0.8		0.8			
Total cover degree	0.3		0.6		0.7			

*The small black circle “∙” indicates the presence of a secondary condition, the big black circle “⚫” indicates the presence of a core condition, the thin circle with × “⊗” indicates the absence of a secondary condition, and the bold circle with × “⊗” indicates the absence of a core condition.*

**TABLE 6:** Matching analysis of school experience and high original innovation personality.

Antecedents	Agreeableness configuration solution	Responsibility configuration solution				Openness configuration solution			
	AS	RS1	RS2	RS3	RS4	OS1	OS2	OS3	OS4
Teacher accomplishments	●	●	●	●		●	●	●	⊗
Democracy teaching	⚫	⚫	⚫	⚫	⚫		⊗		●
Open teaching	⊗	●	●		●	⚫	⚫	⚫	●
Teacher-student relationship	●	●		●	●		●	●	⊗
Peer support	●	●	●	●	●	●		●	⚫
Education level	●		●	●	●	⚫	⚫	⚫	●
Consistency	0.8	0.8	0.8	0.8	0.8	0.9	0.9	0.9	0.9
Original cover degree	0.3	0.4	0.5	0.4	0.4	0.6	0.2	0.4	0.2
Unique cover degree	0.3	0.02	0.1	0.03	0.02	0.1	0.01	0.02	0.1
Total consistency	0.8	0.8				0.9			
Total cover degree	0.3	0.5				0.7			

*The small black circle “∙” indicates the presence of a secondary condition, the big black circle “⚫” indicates the presence of a core condition, the thin circle with × “⊗” indicates the absence of a secondary condition, and the bold circle with × “⊗” indicates the absence of a core condition.*

**TABLE 7:** Matching analysis of work experience and high original innovation personality.

Antecedents	Responsibility configuration solution	Openness configuration solution	
	RW	OW1	OW2
Organizational support	⚫		⊗
Colleague relations	⊗	⊗	
Consistency	0.8	0.9	0.9
Original cover degree	0.3	0.6	0.2
Unique cover degree	0.3	0.1	0.01
Total consistency	0.3	0.9	
Total cover degree	0.8	0.7	

*The small black circle “∙” indicates the presence of a secondary condition, the big black circle “⚫” indicates the presence of a core condition, the thin circle with × “⊗” indicates the absence of a secondary condition, and the bold circle with × “⊗” indicates the absence of a core condition.*

**TABLE 8:** Matching analysis between family experience and non-high original innovation personality.

Antecedents	Agreeableness configuration solution			
	AS1	AS2	AS3	AS4
Family democracy	⊗	⊗	⊗	●
Family size	⊗		●	⊗
Family function	⊗	⊗	⊗	⊗
Family status		●	⊗	●
Family support	⊗	⊗	●	●
Consistency	0.9	0.9	0.9	0.9
Original cover degree	0.3	0.2	0.2	0.2
Unique cover degree	0.04	0.02	0.01	0.1
Total consistency	0.8			
Total cover degree	0.4			

*The small black circle “∙” indicates the presence of a secondary condition, the big black circle “⚫” indicates the presence of a core condition, the thin circle with × “⊗” indicates the absence of a secondary condition, and the bold circle with × “⊗” indicates the absence of a core condition.*

**TABLE 9:** Matching analysis of school experience and non-high original innovation personality.

Antecedents	Extroversion configuration solution		Agreeableness configuration solution		Neuroticism configuration solution	
	ES1	ES2	AS1	AS2	NS1	NS2
Teacher accomplishments	⊗	⊗	⊗	⚫	⊗	⊗
Democracy teaching	⊗	⊗	⊗	⊗	⊗	⊗
Open teaching	⊗	⊗	⊗	⊗	⊗	⊗
Teacher-student relationship	⊗	⊗	⊗	●	⊗	⊗
Peer support	⊗	⚫	⚫	●	⊗	
Education level	⊗	⚫	⚫	⊗		●
Consistency	0.9	0.9	0.9	0.9	0.9	0.9
Original cover degree	0.3	0.2	0.2	0.2	0.2	0.2
Unique cover degree	0.4	0.1	0.1	0.04	0.3	0.03
Total consistency	0.8		0.92		0.2	
Total cover degree	0.3		0.23		0.9	

*The small black circle “∙” indicates the presence of a secondary condition, the big black circle “⚫” indicates the presence of a core condition, the thin circle with × “⊗” indicates the absence of a secondary condition, and the bold circle with × “⊗” indicates the absence of a core condition.*

**TABLE 10:** Matching analysis of work experience and non-high original innovation personality.

Antecedents	Agreeableness		Neuroticism	
	configuration solution		configuration solution	
	AW1	AW2	NW1	NW2
Organizational support		⊗		⊗
Colleague relations	⊗		⊗	
Consistency	0.8	0.8	0.8	0.8
Original cover degree	0.4	0.6	0.4	0.5
Unique cover degree	0.1	0.2	0.1	0.2
Total consistency	0.7		0.8	
Total cover degree	0.7		0.6	

*The bold circle with × “⊗” indicates the absence of a core condition.*

#### Configuration Analysis of High Personality Traits

##### Configuration of High Original Innovation Personality in Family Experience

Table 5 shows that the configuration’s consistency of high extroversion, responsibility, and openness was 0.8, respectively, which means that configurations cover most samples.

###### Family Driving of High Extroversion

According to Table 5, the two configurations both included the core condition “family support” and “family function,” which meant that family support ensured an individual’s continuous attention, perseverance, and gratitude (Tennent and Berthelsen, 1997; Hensley, 2009). This study summarizes how other combinations of conditions activate high extroversion besides the core condition: (1) Effect of family size (family democracy × family size × family function × family status × family support): a large family size weakens the undemocratic control from the parents (Henderson, 2013), and the poor family status focuses on resource exchange and information interaction between individuals and compatriots. (2) Effect of family democracy and status (family democracy × ∼family size × ∼family function × family status × family support). Family with high status and democracy, coupled with small-scale family, helps parents to provide sufficient material support and spiritual guidance for an individual’s outward thinking (Henderson, 2013). The above two configurations indicated that regardless of whether the family status is high or not, it plays an auxiliary role in high extroversion. Family democracy with high or non-high levels produces high extroversion. Non-high democracy enables individuals to seek contact from outside and high social maturity (Turner et al., 2009); high or non-high family size also has an important influence on extroversion. A larger family size compensates for the deficiency of family democracy and status, and more brothers and sisters can gather resources and learn to share with each other, which is helpful for individuals to explore the outside world. On the contrary, small family size, high family status, and family democracy lead individuals to explore the outside world.

###### Family Driving of High Responsibility

According to Table 5, high family status and high family support are both core conditions in the two configurations, which partially verifies that high family status shapes an individual’s social value and principles (Conger and Donnellan, 2007). High family support and high family democracy predict responsibility (Lancee, 2018; Bose and Pal, 2019). In addition to the dual driving mode of family status and family support, this study summarizes the path in which other conditions acted on high responsibility: (1) The effect of democracy: family democracy × ∼family size × family status × family support. This showed that when high family democracy plays an important role, both high family function and non-high family function can produce high responsibility. (2) Effect of size and function: family democracy × family size × family function × family status × family support. Whether family democracy is high or not, high family size and high family function can bring high responsibility. The above configurations showed that sometimes family democracy and family function have nothing to do with responsibility. If the family function is not perfect, when the family is democratic and in a good position, the individual will get some help and acquiescence to produce responsibility. When family democracy is uncertain, other factors with high levels still promote responsibility.

###### Family Driving of High Openness

As shown in Table 5, the configuration solution of high openness is summed up by the four following driving modes according to the core condition: (1) Status driving: family democracy × family function × family status × family support. Family status compensates for the shortcomings of other factors and brings about high openness. (2) Democracy driving: family democracy × ∼family size × family status × family support, family democracy × family size × family function × family support. Regardless of whether the family status is superior or not, other factors such as the secondary condition can produce high openness. Regardless of whether the family function is superior or not, a large family’s status and support can produce high openness. (3) Size driving: family democracy × family size × family status × family support × family function. A large family size and support for individuals can produce high openness when other factors are lacking. Family status as the only index of high level still produces high openness, and even though other conditions are not high, parents with high education and job income have time and energy to teach their children (Husain et al., 2019) and to enhance individual’s motivation for further study (Rothenberg, 2005). When other conditions are not high, high family democracy is expected to produce high openness (Fan and Zhang, 2014). It should also be noted that high family status is not a necessary index to influence high openness, and original innovation talent comes from a humble family (Kauffman and Kauffman, 2011; Silva-Fisher et al., 2020).

##### Configuration of High Original Innovation Personality in School Experience

###### School Driving of High Agreeableness

As shown in Table 6, democracy in higher education had an important impact on high agreeableness, which is consistent with the view of Tummala-Narra’s emphasis on democracy, contributing to the trust of teachers and students (Tummala-Narra, 2009; Glaser, 2010). A non-high level of open teaching played an important role in high agreeableness, because open classrooms encourage the collision of ideas and heated debates among individuals, as in Tummala Narra’s opinion.

###### School Driving of High Responsibility

As shown in Table 6, high democracy teaching played an important role in high responsibility, while other conditions with high levels were used as the secondary conditions to predict high responsibility. Just as Pinson mentioned, students are eager to give themselves a sense of self-affirmation, and democratic teaching makes students more confident and independent (Pinson et al., 2020). All configurations indicated that high responsibility was driven by high democracy teaching and also showed that just one fragile condition emerging among teacher accomplishment, educational level, and open teaching could produce high responsibility.

###### School Driving of High Openness

As shown in Table 6, according to the core conditions, two types of driving forces were summarized as follows: Open teaching driving and peer support driving. Whether democratic teaching is superior or not, high teacher accomplishment, high educational level, and high open teaching can bring openness; peers replace the teacher’s role in the case that teacher accomplishment and teacher-student relationships are both non-high, relying on peers and using the curriculum could still bring openness. Open teaching plays an important role in high responsibility and openness, but it cannot be the core condition for high agreeableness. High democracy teaching has an important influence on high agreeableness and responsibility. High open teaching and educational level play an important role in high openness.

##### Configuration Solution of High Original Innovation Personality in Working Experience

###### Work Driving of High Responsibility

As shown in Table 7, high organizational support had an important influence on high responsibility, which is similar to Hull’s research (Hull, 1978). Organizations tend to choose individuals with similar goals, and in turn, individuals who perceive organizational support make employees care about the organization’s benefit, strengthen their sense of mission to help realize the organization’s objectives, and work hard to provide feedback to the organization (Law et al., 2007). The support of an organization’s economic and social resources effectively enhances the employees’ autonomy, sense of competence, and satisfaction. After being motivated, employees will be more careful, devote more energy to work, set challenging goals, achieve self-growth, and be more willing to work in a team. The positive effect of a high colleague relationship on high responsibility has not been observed.

###### Work Driving of High Openness

As shown in Table 7, non-high organizational support had a more important effect on high openness. The reason may be that creative individuals are unique in the work environment, often act contrary to the organization’s goals, and do not follow the rules, but it may lead to unexpected achievements.

#### Configuration Analysis of Non-high Personality Traits

##### Configuration of Non-high Original Innovation Personality in Family Experience

As shown in Table 8, non-high agreeableness was mainly driven by non-high family function, and there were three paths. (1) “ ∼family democracy × ∼family size × ∼family function × ∼family support” shows that non-high agreeableness is mostly derived from a bad family experience. Furthermore, non-high family democracy and non-high family support of small-size families have a great impact on individuals as well as a non-ideal family education, which is easy to create individuals’ lack of agreeableness. Uncaring parents make individuals isolated and suspicious. This configuration also suggests that other conditions with non-high levels lead to less agreeableness regardless of the level of family status. (2) “family democracy × family function × family status × family support” indicates that no matter the size of the family, it shows that family status is acceptable but lacks democracy and support, especially family function, which make it easy to bring non-high agreeableness. (3) “family democracy × family size × ∼family function × ∼family status × family support” shows that a large-size family pays attention to family support and a lack of family democracy, in addition to the general status of the family, easily causes non-high agreeableness amongst individuals.

##### Configuration Solution of Non-high Original Innovation Personality in School Experience

###### Non-high Extroversion Caused by School

As shown in Table 9, a non-high teacher-student relationship was the core condition of non-high extroversion. The configuration solution was driven by a non-high teacher-student relationship, which was mainly divided into the three following paths: the core function of non-high educational level, the core function of non-high teacher accomplishments, and the core function of non-high level of open teaching. In the former form, the poor teacher-student relationship and the poor educational level have an important effect on the non-high extroversion, while the other factors have minor effects, and the poor teacher-student relationship has an impact on the students’ curiosity. The second type showed that inadequate teacher accomplishments and closed teaching have an important impact on non-high extroversion. Moreover, especially high peer support and educational level may aggravate the individual’s perception of inadequate teacher accomplishments and open teaching, thus reducing the external thirst for knowledge. In addition, it is unfavorable for high extroversion when other factors are poor, and the individual is less willing to replace their knowledge system even if their educational level and peer influence are good.

###### Non-high Agreeableness Caused by School

As shown in Table 9, non-high democracy teaching and open teaching had an auxiliary effect on non-high agreeableness. High peer support had different effects on non-high agreeableness, which might be related to the fact that researchers paid more attention to the maintenance of peers. There were two kinds of driving modes, namely, lack of teacher accomplishments and lack of a high educational level. The former and the latter have the reverse effect of teacher accomplishments and educational level, which easily lead to individuals’ non-high agreeableness.

###### Non-high Neuroticism Caused by School

If the teacher accomplishments, democracy teaching, open teaching, and teacher-student relationship are at a non-high level, they have a core or secondary impact on non-high neuroticism. The lack of both teaching accomplishments and open teaching is especially likely to lead to non-high neuroticism when other factors are not high.

##### Configuration Solution of the Working Experience of Non-high Original Innovation Personality

As shown in Table 10, the lack of any condition in the working experience might lead to non-high agreeableness or non-high neuroticism. For example, individuals become difficult to get along with during a lack of high-intensity work or crisis awareness and eventually exhibit a laissez-faire attitude and relax.

## Conclusion

First, the family driving of high extroversion can be divided into size driving, democracy driving, and status driving under the premise of high family support and non-high family function. Second, on the premise of high family status and family support, the family driving of high responsibility can be divided into high-democracy driving, high-scale driving and high-function driving, and school driving, which can be shown as high-teaching democracy driving. Workplace driving mainly involved high organizational support. Third, family driving of high openness was divided into high family status driving, high family democracy driving, and high family size driving. School driving included both high open teaching and educational level driving and high peer driving. Workplace driving was non-high organizational support driving and non-high colleague relationship driving. Fourth, agreeableness was high democracy teaching driving and non-high level of open teaching driving. Finally, there was an asymmetric causal relationship between high or non-high original innovation personality configuration effects. The following different views were obtained: high or non-high family status may bring high extroversion, high openness, high responsibility comes from different family sizes, high agreeableness was related to non-high level of open teaching, non-high agreeableness was related to teacher accomplishments and educational level, high peer support, and non-high family function. Non-high teacher accomplishments and teacher-student relationship may produce high openness; Non-high extroversion came with the relationship between teachers and students. Lack of both teacher accomplishments and open teaching led to high neuroticism, especially when other factors were not high.

### Research Implications

Considering that one dimension with difference changes the whole early growth process, the influence of multiple growth experiences on personality is complex, and the causes of high and non-high personality traits cannot be reversed. Accordingly, we regarded the early growth experience as a whole, took the early growth experience as the antecedent of personality, and constructed the model by integrating the early growth theory with the personality trait theory to discover multiple and complex causality relationships between condition configurations made up of various early growth experiences and personality traits, in order to ensure conclusion universality. We exactly discovered that the same level of personality depends on the configuration that consists of various experiences, rather than a certain experience. A certain experience may produce different levels of a certain personality. The multiple and complex causality relationships exist between condition configurations made up of various early growth experiences and personality traits, which enlighten us on how to strengthen the positive effects or avoid the negative effects from the early growing-up experiences on original innovation talents’ personality traits in order to provide practical inspiration for the training of original innovative talents.

### Suggestions

#### Give Play to the Core Advantages of Early Growth Factors

(1) Families should attach importance to the effect of high democratic parenting on high openness. By virtue of family status, democracy should be promoted, and individual extroversion should be encouraged. (2) Families should give priority to the role of family socioeconomic status in the cultivation of individual social concepts, handling of affairs, and national consciousness (Bryant et al., 2006). (3) Families should provide emotional support or assistance when individuals yearn for the outside world or overcome bottlenecks, satisfy their thirst for knowledge, and make them have positive emotions and responsibilities, especially in large-scale families (Tennent and Berthelsen, 1997). (4) Teachers should advocate democracy in teaching, which helps individuals to be honest and modest, to dare to question knowledge, to have the host consciousness in class, to remove prejudice, to reach a consensus, to enhance mutual trust, to improve individual self-esteem and self-confidence, and to be more enthusiastic, independent, and patriotic. (5) Schools should encourage the improvement of teaching content design, discussion teaching, and so on to achieve individual ideological liberation and encourage individuals to continue studying to broaden their horizons. The open evaluation of teachers only positions in scores, which makes students no longer study utilitarianism and explore the outside world. With the individual knowledge base and depth optimization, there are more frontier judgments to the outside world.

#### Avoid Adverse Effects of Growing-Up Experience on Personality

(1) Large wealthy families’ resources are weakly diluted among their children. However, large poor families have insufficient functions so that the parents have no time or money to invest in individual education, but they expect their children to achieve and provide their siblings with emotional support; therefore, individuals are probably highly extroverted and open. (2) Parents provide emotional support when individuals face difficulties, use family resources to serve individual development, and individuals regain confidence and release the suppressed gift by taking advantage of family status in shaping external relations. (3) Excessive education easily leads to rigid thinking and dependence. Even if some cases of high personality have a non-high family function, they have a high level of family size, family status, and family support, so they still have high openness, high extroversion, and high responsibility. (4) In debates, everyone will be questioned by others (Husain et al., 2019); thus, mutual respect and understanding may be ignored, which can easily educate harsh, silent, unfriendly, and uncooperative individuals. Schools should advocate open teaching that pays attention to emotions and respect. (5) Teacher accomplishments cannot meet an individual’s needs for knowledge, and the relationship between teachers and students is not harmonious, in which peer support should be an individual’s most likely way to seek support and to improve learning ability. (6) Teaching democracy that allows students to identify or reject certain knowledge should not ignore authority; it needs to guide students’ rationality and prevent adverse effects from the rigidity of democracy and unconsciousness for openness. (7) Individuals in small families are more confident at an early age, owing to attention and praise, but they may lack willpower. Individuals with siblings are easily neglected by parents, but some original innovation talents are still concerned with big families whose resources are limited because parents persist in hard training, so that an individual treasures chance, shoulders responsibility, works hard, and gives back to their family. (8) Organizational support and organizational cohesion must be strengthened simultaneously. Employees will stimulate work enthusiasm, achievement motivation, responsibility sense, and the confidence to face difficulties and reduce pressure.

## Data Availability Statement

The original contributions presented in the study are included in the article/supplementary material, further inquiries can be directed to the corresponding author.

## Ethics Statement

Ethical review and approval was not required for the study on human participants in accordance with the local legislation and institutional requirements. The Ethics Committee waived the requirement of written informed consent for participation.

## Author Contributions

LZ: conceptualization, methodology, software, and writing-reviewing and editing. YW: writing-reviewing and editing, supervision. DY: investigation, data curation, and validation. YL and WL: writing-original draft preparation. All authors contributed to the article and approved the submitted version.

## Conflict of Interest

The authors declare that the research was conducted in the absence of any commercial or financial relationships that could be construed as a potential conflict of interest.

## Publisher’s Note

All claims expressed in this article are solely those of the authors and do not necessarily represent those of their affiliated organizations, or those of the publisher, the editors and the reviewers. Any product that may be evaluated in this article, or claim that may be made by its manufacturer, is not guaranteed or endorsed by the publisher.

## References

[B1] Abbott A. (2006). Croatian scientists call for openness over funding. *Nature* 439:7. 10.1038/439007a 16397469

[B2] Averill J. R. Chon K. K. Hahn D. W. (2001). Emotions and creativity. *East West* 4 165–183. 10.1111/1467-839x.00084

[B3] Barron F. (1969). *Creative Person and Creative Process.* New York, NY: Holt, Rinehart and Winston.

[B4] Bartneck. (2008). The asymmetry between discoveries and inventions in the nobel prize in physics. *Tech. Arts* 6 73–77. 10.1386/tear.6.1.73/1

[B5] Beard G. M. (2012). *Legal Responsibility in Old Age: Based on Researches into the Relation of Age to Work.* Charleston, SC: Nabu Press.

[B6] Bose S. Pal D. (2019). Impact of employee demography, family responsibility and perceived family support on workplace resilience. *Glob. Busi. Rev.* 21 1249–1262. 10.1177/0972150919857016

[B7] Brockelman W. Y. (2007). How to produce a scientist. *ScienceAsia* 33 367–369. 10.2306/scienceasia1513-1874.2007.33.367

[B8] Bronfenbrenner U. (1986). Development in context: paradox sans paradigm. *PsycCRITIQUES* 31 527–528. 10.1037/024900

[B9] Bryant B. K. Zvonkovic A. M. Reynolds P. (2006). Parenting in relation to child and adolescent vocational development. *J. Vocat. Behav.* 69 149–175. 10.1016/j.jvb.2006.02.004

[B10] Chauhan A. Goel M. Arora R. G. (2018). Impact of organisational variables on higher education academicians. *Asian J. Manag.* 9 1259–1272. 10.5958/2321-5763.2018.00201.9

[B11] Clark R. D. Rice G. A. (2010). Family constellations and eminence: the birth orders of nobel prize winners. *J. Psychol.* 110 281–287. 10.1080/00223980.1982.9915350

[B12] Clarke T. E. (2002). Unique features of an R&D work environment and research scientists and engineers. *Knowl. Technol. Policy* 15 58–69. 10.1007/s12130-002-1005-1

[B13] Conger R. D. Donnellan M. B. (2007). An interactionist perspective on the socioeconomic context of human development. *Annu. Rev. Psychol.* 58 175–199. 10.1146/annurev.psych.58.110405.085551 16903807

[B14] Corwyn R. F. Bradley R. H. (2002). Stability of maternal socioemotional investment in young children. *Parenting* 2 27–46. 10.1207/S15327922PAR0201_2

[B15] Csikszentmihalyi M. (2010). *The Systems Model of Creativity.* Dordrecht: Springer Netherlands, 10.1002/9781118367377.ch25

[B16] Dacey J. S. Lennon K. H. (1998). *Understanding Creativity: The Interplay of Biological, Psychological, and Social Factors.* Hoboken, NJ: John Wiley & Sons Inc.

[B17] Fan J. Zhang L.-F. (2014). The role of perceived parenting styles in thinking styles. *Learn. Indiv. Differ.* 32 204–211. 10.1016/j.lindif.2014.03.004

[B18] Glaser B. G. (2010). Attraction, autonomy, and reciprocity in the scientist - supervisor relationship. *Ground. Theory Rev.* 9 1–19.

[B19] Guilford J. P. (1968). *Intelligence, Creativity and Their Educational Implications.* San Diego, CA: RR Knapp.

[B20] Harrington D. M. (2011). “Creative environments, conditions, and settings,” in *Encyclopedia of Creativity*, eds Runco M. A. Pritzker S. R. (Amsterdam: Elsevier), 264–272. 10.1016/B978-0-12-375038-9.00043-1

[B21] He P. Sun R. Zhao H. Zheng L. Shen C. (2020). Linking work-related and non-work-related supervisor-subordinate relationships to knowledge hiding: a psychological safety lens. *Asian. Bus. Manag.* 10.1057/s41291-020-00137-9

[B22] He P. Jiang C. Xu Z. Shen C. (2021). Knowledge hiding: current research status and future research directions. *Front. Psychol.* 12:748237. 10.3389/fpsyg.2021.748237 34777143 PMC8586422

[B23] Henderson E. M. (2013). *Family Size and Educational Consequences in the Uk.* PhD Thesis, Oxford: Oxford University.

[B24] Hensley E. W. (2009). *Nobel Laureates: Their Parents’ Influence.* Parenting for High Potential, 1–3.

[B25] Hull D. L. (1978). Altruism in science: a sociobiological model of co-operative behaviour among scientists. *Anim. Behav.* 26 685–697. 10.1016/0003-3472(78)90135-5

[B26] Husain N. I. A. E. Meisenberg G. Becker D. Bakhiet S. F. Essa Y. A. S. Lynn R. (2019). Intelligence, family income and parental education in the sudan. *Intelligence* 77:101402. 10.1016/j.intell.2019.101402

[B27] Kaewviset S. Nongnuch P. Wannapa S. Suthiluk N. Kanyaratt S. (2015). Inspired by the nobel laureates: a typical event-based inspirational motivation in science education. *Adv. Sci. Lett.* 21 2425–2428. 10.1166/asl.2015.6298

[B28] Kapranov S. (2019). Japanese nobel winners. *Chinese Stud.* 2019 61–70. 10.15407/chinesest2019.02.061

[B29] Kauffman G. B. Kauffman L. M. (2011). The road to stockholm: nobel prizes. *Sci. Sci. Chem. Int.* 25:24. 10.1515/ci.2003.25.1.24

[B30] Kempel C. R. (2009). *The Personality and Mental Health of Physical and Social Scientists.* Dissertations & Theses Gradworks. Avaliable online at: https://www.proquest.com/dissertations-theses/personality-mental-health-physical-social/docview/205444987/se-2?accountid=130565 (accessed March 15, 2020).

[B31] Lancee B. (2018). The economic returns of immigrants’ bonding and bridging social capital: the case of the Netherlands. *Int. Migrat. Rev.* 44 202–226. 10.1111/j.1747-7379.2009.00803.x

[B32] Law K. S. Wong C.-S. Huang G.-H. Li X. (2007). The effects of emotional intelligence on job performance and life satisfaction for the research and development scientists in China. *Asia Pacific J. Manag.* 25 51–69. 10.1007/s10490-007-9062-3

[B33] Lee S. M. Daniels M. H. Kissinger D. B. (2006). Parental influences on adolescent adjustment: parenting styles versus parenting practices. *Family J.* 14, 253–259. 10.1177/1066480706287654

[B34] National Research Council, Division on Engineering and Physical Sciences, Standing Committee on Technology Insightâ¬”Gauge, Evaluate, Review, Committee on Global Science and Technology Strategies (2010). *S&T Strategies of Six Countries: Implications for the United States.* Washington, DC: National Academies Press, 10.17226/12920

[B35] Pereira A. I. F. Canavarro C. Cardoso M. F. Mendonça D. (2008). Patterns of parental rearing styles and child behaviour problems among portuguese school-aged children. *J. Child Fam. Stud.* 18 454–464. 10.1007/s10826-008-9249-3

[B36] Pinson H. Meshulam A. Michlin Y. (2020). Can teachers disrupt their professional identity and enable children’s participation? Comparing teachers’ and municipal officials’. Work with students in democratic spaces. *Teach. Teach. Educ.* 96:103178. 10.1016/j.tate.2020.103178

[B37] Reid S. E. Brentani U. D. Kleinschmidt E. J. (2014). Divergent thinking and market visioning competence: an early front-end radical innovation success typology. *Indus. Mark. Manag.* 43 1351–1361. 10.1016/j.indmarman.2014.08.011

[B38] Rothenberg A. (2005). Family background and genius Ii: nobel laureates in science. *Can. J. Psychiatry* 50 918–925. 10.1521/bumc.69.1.81.62268 16494261

[B39] Saether E. A. (2019). Motivational antecedents to high-tech R&D Employees’ innovative work behavior: self-determined motivation, person-organization fit, organization support of creativity, and pay justice. *J. High Technol. Manag. Res.* 30:100350. 10.1016/j.hitech.2019.100350

[B40] Sen R. S. Sharma N. (2013). The familial context of creativity: patterns of nurturance in families of creative children. *Psychol. Stud.* 58 374–385. 10.1007/s12646-013-0221-y

[B41] Silva-Fisher J. Nervo L. M. Addo T. Guerra A. Odinammadu K. (2020). Voices on diversity: multiple paths to becoming a scientist. *Mol. Cell* 80 752–757. 10.1016/j.molcel.2020.11.021 33275884

[B42] Silvanto S. Ryan J. (2018). An investigation into the core appeals for nation branding to attract and retain talent to enhance a country’s competitiveness. *Compet. Rev.* 28 584–604. 10.1108/cr-05-2017-0036

[B43] Simonton D. K. (1984). Artistic creativity and interpersonal relationships across and within generations. *J. Person. Soc. Psychol.* 46 1273–1286. 10.1037/0022-3514.46.6.1273

[B44] Southerland S. A. Bahbah S. U. (2011). “Educational policy of accountability and women’s representation in science,” in *Celebrating the 100th Anniversary of Madame Marie Sklodowska Curie’s Nobel Prize in Chemistry*, (Rotterdam: Sense Publishers).

[B45] Sternberg R. J. Lubart T. I. (1993). Investing in creativity. *Psychol. Inq.* 4 229–232. 10.1207/s15327965pli0403_16

[B46] Su X. (2009). Postdoctoral training, departmental prestige and scientists’. Research productivity. *J. Technol. Trans.* 36 275–291. 10.1007/s10961-009-9133-3

[B47] Tennent L. Berthelsen D. (1997). Creativity: what does it mean in the family context? *J. Austr. Res. Early Childh. Educ.* 1 91–104.

[B48] Thomas E. Romero M. R. Billia A. R. Osman A. G. (2016). Expanding the therapeutic spectrum of artemisinin:activity against infectious diseases beyond malaria and novel pharmaceutical developments. *World J. Tradit. Chin. Med.* 2 5–27. 10.15806/j.issn.2311-8571.2016.0002

[B49] Torrance E. P. (1962). *Guiding Creative Talent.* Englewood Cliffs, NJ: Prentice-Hall, Inc.

[B50] Tummala-Narra P. (2009). Teaching on diversity: the mutual influence of students and instructors. *Psychoanal. Psychol.* 26 322–334. 10.1037/a0016444

[B51] Turner E. A. Chandler M. Heffer R. W. (2009). The influence of parenting styles, achievement motivation, and self-efficacy on academic performance in college students. *J. Coll. Stud. Dev.* 50 337–346. 10.1353/csd.0.0073 34409987

[B52] Verganti R. (2008). Design, meanings, and radical innovation: a metamodel and a research agenda. *J. Prod. Innov. Manag.* 25 436–456. 10.1111/j.1540-5885.2008.00313.x

[B53] Wang H. (2013). *China’s Competition for Global Talents: Strategy, Policy and Recommendations.* Rochester, NY: Social Science Electronic Publishing, 1–19. 10.2139/ssrn.2263701

[B54] Youtie J. Rogers J. Heinze T. Shapira P. Tang L. (2013). Career-based influences on scientific recognition in the united states and europe: longitudinal evidence from curriculum vitae data. *Res. Policy* 42 1341–1355. 10.1016/j.respol.2013.05.002

[B55] Zuckerman H. (1977). The upward path. (Book Reviews: Scientific Elite. Nobel Laureates in the United States). *Science* 196 754–755. 10.1126/science.196.4291.754 17776883

